# Facile Engineering of CoS@NiS Heterostructures for Efficient Oxygen Evolution Reaction

**DOI:** 10.3390/nano15161216

**Published:** 2025-08-08

**Authors:** Ting Yang, Aiyi Dong, Weimin Liao, Xun Zhang, Yinhua Ma, Li Che, Honglin Gao

**Affiliations:** 1 Transportation Engineering College, Dalian Maritime University, Dalian 116026, China; yangting@dlmu.edu.cn (T.Y.); zx2220223740@dlmu.edu.cn (X.Z.); 2 School of Science, Dalian Maritime University, Dalian 116026, China; liche@dlmu.edu.cn; 3 Marine Engineering College, Dalian Maritime University, Dalian 116026, China; lwm20021202@dlmu.edu.cn

**Keywords:** water splitting, OER, electrocatalysts, CoS@NiS, heterogeneous interface

## Abstract

Hydrogen production by the electrolysis of water has become an important way to prepare green hydrogen because of its simple process and high product purity. However, the oxygen evolution reaction (OER) in the electrolysis process has a high overpotential, which leads to the increase of energy consumption. Developing efficient, stable and low-cost electrolytic water catalyst is the core challenge to reduce the reaction energy barrier and improve the energy conversion efficiency. CoS@NiS-80% nanosheets with rich heterogeneous interfaces were successfully synthesized by hydrothermal reaction and sulfuration. Heterogeneous interface not only promotes the effective charge transfer between different materials and reduces the charge transfer resistance but also accelerates the four-electron transfer process through the synergistic effect of nickel and cobalt atoms. Under alkaline conditions, the overpotential of CoS@NiS-80% nanosheets was only 280 mV at a current density of 10 mA cm^−2^, with a Tafel slope of 100.87 mV dec^−1^. Furthermore, it could work continuously for 100 h, exhibiting its outstanding stability. This work provides a novel approach for improving the OER performance of transition metal sulfide-based electrocatalysts through heterogeneous interface engineering.

## 1. Introduction

With the transformation of the global energy structure to clean and low carbon, hydrogen energy, as a secondary energy with high energy density and zero carbon emission, is regarded as one of the key carriers to achieve the goal of carbon neutrality [1,2]. Hydrogen production by the electrolysis of water has become an important way to prepare green hydrogen because of its simple process and high product purity [3,4]. However, the hydrogen evolution reaction (HER) and oxygen evolution reaction (OER) in the electrolysis process have high overpotential, which leads to the increase of energy consumption and restricts the large-scale application of this technology [5,6,7]. Developing efficient, stable and low-cost electrolytic water catalyst is the core challenge to reduce the reaction energy barrier and improve the energy conversion efficiency [8,9]. In recent years, precious metal-based materials (such as Pt/C and RuO_2_) have been regarded as benchmark catalysts because of their excellent catalytic activity, but their scarcity and high cost limit their industrial promotion [10,11]. Therefore, to realize the large-scale electrolysis of water to produce hydrogen, highly efficient and durable non-noble metal-based electrocatalysts are needed to explore [12,13].

In recent years, low-cost transition metal-based electrocatalysts have made continuous progress [14], which can be simply divided into oxides [15,16], phosphides [17] and sulfides etc. Transition metal chalcogenides have attracted much attention due to their adjustable electronic structure, controllable morphology, rapid electron transport capacity and good catalytic activity [7,18,19,20,21]. For instance, Wang et al. synthesized CeO_2_/NiCo_2_S_4_ by hydrothermal synthesis. CeO_2_/NiCo_2_S_4_ only needs 271 mV to drive the current density of 100 mA cm^−2^ [22]. Zhang et al. synthesized CoS_2_/MoS_2_/Ni_3_S_2_/NF, which needed 200 mV to reach 10 mA cm^−2^ [23]. Among transition metal sulfides, CoS and its derivatives (such as NiCo_2_S_4_) are one of the most promising OER catalysts by virtue of their comprehensive advantages of activity, stability and conductivity [20,24]. The Co^2+^/Co^3+^ redox pair exhibits high intrinsic activity in OER, especially the dynamically generated CoOOH surface layer (real active phase), which can effectively catalyze OER [25,26,27]. Additionally, the CoOOH surface layer inhibits the corrosion of alkaline environments to the catalyst. The energy band structure of CoS is close to that of metal, which is suitable for high-current-density operation. Although great efforts have been made so far, transition metal chalcogenides (TMCs) still have some problems, such as insufficient active sites, insufficient stability and poor hydrophilicity [28]. Interface engineering (such as the construction of heterogeneous) is usually adopted to optimize the electronic structure and active site exposure of TMCs so that the OER performance can be further improved [29,30,31]. Interface engineering can adjust and control the electronic structure of the catalyst, such as the d-band structure and O p-band, thus optimizing the adsorption and desorption energy of intermediates to reduce the reaction energy barrier [24,32]. Consequently, significant synergistic effects are observed, improving the OER performance of TMCs. Actually, CoS is often combined with other sulfides to break through the limitation of a single component by interface engineering [33,34]. Luo et al. synthesized an amorphous/crystalline heterostructure catalyst (a-CoS/Ni_3_S_2_) by the hydrothermal method. It exhibited excellent OER activity due to its rich heterogeneous interfaces [35]. Feng et al. reported cactus-like NiCo_2_S_4_@NiFe LDH hollow spheres, and the catalyst exhibited enhanced OER performance [36]. In recent years, the heterostructure of layered and 2D materials has been widely reported due to its adjustable electronic, optical and mechanical properties at the atomic scale [37,38].

There are previous studies proving that the surface reconstruction of TMCs inevitably takes place in the OER process. Surface reconstruction changes TMCs catalysts in both composition and structure. The transformation brings both advantages and disadvantages to the OER performance of TMCs. Under high OER overpotential, the chemical band between S and the transition metal atom is facile to break [39,40]. Transition metal oxyhydroxides with higher OER activity will be formed on the catalyst surface, thus greatly increasing the number of active sites and effectively reducing the reaction energy barrier. On the contrary, the transition metal oxyhydroxides will diminish the conductivity of TMCs [41]. Additionally, surface reconstruction will weaken the adhesion between catalyst and substrate, which may lead to the dissolution of catalyst [42]. Optimizing the composition and structure of TMCs is pivotal to make the surface reconstruction favorable to the OER reaction [43,44].

In light of the above discussion, CoS@NiS-x nanosheets with rich heterogeneous interfaces were synthesized via hydrothermal sulfurization. The CoS@NiS-x is a catalyst formed by the combination of CoS and NiS. There are sufficient heterogeneous interfaces in the nanosheets of CoS@NiS-x. Sufficient heterogeneous interfaces and the synergistic effect between Co atoms and Ni atoms can optimize the electronic structure of catalysts. Changing the CoS and NiS ratio allows the catalysts’ electronic structure to be readily adjusted to achieve the optimal one. Rich heterogeneous interfaces not only promote the effective charge transfer between different materials, reducing the charge transfer resistance, but also improve the stability of CoS. In addition, the surface of TMCs will be reconstructed during OER, and high-activity transition metal hydroxyl oxides will be formed. Under the combined effects of surface reconstruction and abundant heterogeneous interfaces, CoS@NiS-80% exhibits relatively good OER performance. In an alkaline environment, the overpotential of CoS@NiS-80% nanosheets is only 280 mV at a current density of 10 mA cm^−2^. Additionally, at a current density of 10 mA cm^−2^, it can work stably for 100 h without obvious performance attenuation. Furthermore, at 80 °C, CoS@NiS-80% exhibits better OER activity, with an overpotential of 201 mV to achieve 10 mA cm^−2^. In alkaline-simulated seawater and alkaline seawater environments, the overpotential of CoS@NiS-80% at 10 mA cm^−2^ is 363 and 402 mV, respectively. This work provides an idea for enhancing the OER performance of transition metal chalcogenide-based electrocatalysts by heterogeneous interface engineering.

## 2. Materials and Methods

### 2.1. Materials

Co(NO_3_)_2_·6H_2_O (Macklin, Shanghai Macklin Biochemical Co., Ltd., Shanghai, China, AR, 99%), Ni(NO_3_)_2_·6H_2_O (Macklin, Shanghai Macklin Biochemical Co., Ltd., Shanghai, China, AR, 99%), urea (Macklin, Shanghai Macklin Biochemical Co., Ltd., Shanghai, China, AR, 99%), ethylene glycol (Macklin, Shanghai Macklin Biochemical Co., Ltd., Shanghai, China, AR, >99%), Na_2_S·9H_2_O (Aladdin, Shanghai Aladdin Biochemical Technology Co., Ltd., Shanghai, China, AR, ≥98%), RuO_2_ (Sinero, Suzhou Sinero Technology Co., Ltd., Suzhou, China, AR, 99%), KOH (Macklin, Shanghai Macklin Biochemical Co., Ltd., Shanghai, China, AR, 99%).

### 2.2. Preparation of CoS, NiS and CoS@NiS-x Nanosheets

For synthesizing CoS nanosheets, 1.25 mmol of Co(NO_3_)_2_·6H_2_O and 5 mmol of urea were dissolved in 17.5 mL of ethylene glycol and 2.5 mL of deionized water to form homogenous pink solution via stirring. Then, the obtained pink solution was transferred into a Teflon-lined stainless-steel autoclave and kept at 120 °C for 4 h. Afterwards, the obtained dark green a-Co(OH)_2_ nanosheets were collected by centrifugation and directly immersed in 30 mL of 1 mol L^−1^ Na_2_S solution for 13 h and stirred at 500 rpm. Ultimately, centrifugation was used to collect the black product, followed by washing with deionized water several times and drying in a vacuum environment of 50 °C for 17 h.

Excluding 1.25 mmol of Co(NO_3_)_2_, whuch was changed to 1.25 mmol of Ni(NO_3_)_2_, the synthesis steps of NiS nanosheets were consistent with CoS.

As for the CoS@NiS-x nanosheets, 1.25 mmol of Co(NO_3_)_2_ was changed to 1.25·x (x = 20%, 50%, 80%) mmol of Ni(NO_3_)_2_·6H_2_O and 1.25·(1 − x) mmol of Co(NO_3_)_2_·6H_2_O. The following preparation steps of CoS@NiS-x nanosheets were consistent with CoS.

### 2.3. Preparation of Electrolyte

- Alkaline-simulated seawater electrolyte

First, we weighed 0.13 mol KOH and 0.65 mol NaCl, and then we measured out 130 mL of deionized water (pH = 8.82). We dissolved the weighed KOH and NaCl in the deionized water. Once the solution has cooled to room temperature, we measured its pH (pH = 14.20). Finally, the cooled solution was used as an electrolyte for an electrochemical test.

b. Alkaline seawater electrolyte

Without pretreatment, we measured 130 mL of natural seawater (pH = 8.30) and directly dissolved 0.13 mol KOH in it. Because of metal ions such as Mg^2+^ in natural seawater, these ions will consume OH^−^ in the electrolyte and form precipitates, resulting in a decrease in the pH of the alkaline seawater electrolyte (pH = 14.06). Therefore, additional KOH is added to the alkaline seawater electrolyte to adjust its pH to be the same as that of a 1 M KOH electrolyte solution. Eventually, the alkaline seawater electrolyte, with a pH of 14.31, can be directly used for electrochemical tests without filtering out the hydroxide precipitates.

### 2.4. Electrochemical Measurements

A CS310MA electrochemical workstation (Wuhan CorrTest) was employed to test the electrochemical data. All the electrochemical measurements were performed under alkaline condition of 1 M KOH. We dispersed 3 mg catalyst and 35 μL Nafion in 1.3 mL dispersing agent, which was prepared by mixing deionized water and isopropanol according to the volume ratio of 3:1. Then, the catalyst ink was homogenized by ultrasonic treatment. And all catalyst ink was loaded onto the clean carbon paper electrode (1 cm × 1 cm) with a loading of 3 mg cm^−2^. The carbon paper electrode with the catalyst was the working electrode in a three-electrode system, while Hg/HgO was the reference electrode and the graphite rod worked as the counter electrode. The inverse scan (potential from high to low) was adopted to avoid oxidation peaks in the linear sweep voltammetry (LSV) [45]. Different scan rates (20, 40, 60, 80, 100, 120 mV s^−1^) were employed to test the electrochemical double-layer capacitance (C_dl_). Electrochemical impedance spectroscopy (EIS) was carried out to assess the dynamics of charge transfer with frequency from 0.01 Hz to 100 kHz. In this article, all voltages will be converted into the values of reversible hydrogen electrodes using the following equation: E (RHE) = E (Hg/HgO) + 0.098 + 0.0592 × pH.

## 3. Results and Discussion

### 3.1. Characterization

**Figure 1:**
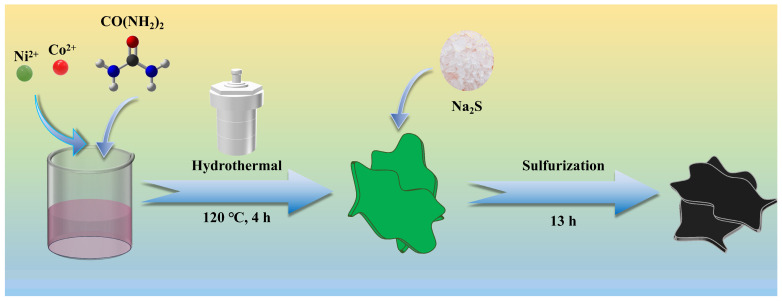
Schematic diagram of the synthesis process of CoS@NiS-x.

As displayed in Figure 1, the CoS@NiS-x was prepared via the hydrothermal process, which exhibited a nanosheet structure. A typical preparation process included two steps. Co(OH)_2_@Ni(OH)_2_-x nanosheets were grown by hydrothermal process. Subsequently, the Co(OH)_2_@Ni(OH)_2_-x nanosheets were immersed in Na_2_S solution. While retaining the original nanosheet structure and enriching the heterogeneous interfaces, S atoms replaced the hydroxyl groups in Co(OH)_2_@Ni(OH)_2_ [46]. Finally, a CoS@NiS-x nanosheet electrocatalyst with rich heterogeneous interfaces was formed. The specific surface area is an important parameter of the electrochemical activity of reactive nanomaterials [47]. The BET nitrogen adsorption and desorption test is used to evaluate surface area, pore size and distribution. The BET surface area value calculated for CoS@NiS-80% nanosheets is 39.1680 m^2^ g^−1^.

**Figure 2:**
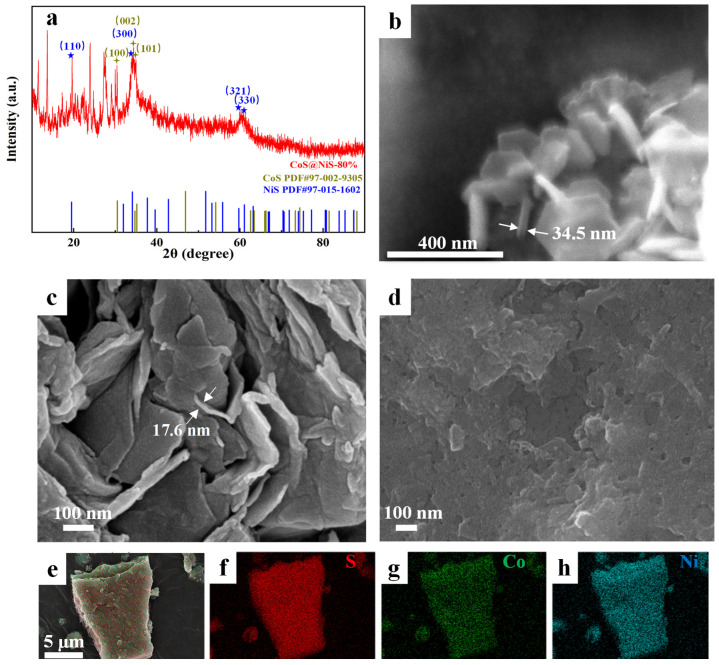
(**a**) The XRD patterns of CoS@NiS-80% (blue represents NiS PDF#97-015-1602, and the positions of some corresponding crystal planes are marked by blue stars; brown represents CoS (PDF#97-002-9305), and the positions of some corresponding crystal planes are marked by brown stars). (**b**) The SEM images of CoS@NiS-20%. (**c**,**d**) The SEM images of CoS@NiS-80% before and after the 100 h OER stability test. (**e**–**h**) Elemental mapping images of CoS@NiS-80% before the electrochemical tests. Red represents S, green represents Co and blue represents Ni.

Figure 2a shows the X-ray diffraction (XRD) pattern of CoS@NiS-80%. Two distinct characteristic peaks in the figure are well matched with the standard diffraction pattern reported for NiS (PDF#97-015-1602). The peaks at 2θ values of 19.5°, 34.1°, 59.7° and 60.1° can be assigned to the (110), (300), (321) and (330) crystal planes of NiS. However, the peaks of CoS are not as obvious as NiS in the figure. Four standard peaks of CoS (PDF#97-002-9305) are marked in the figure. These standard peaks are found at 30.5°, 34.7°, and 35.2° and corresponding to the (100), (002) and (101) crystal planes, respectively. XRD results show that CoS and NiS coexist in the sample. Furthermore, the structural characteristics of the sample are closer to NiS. This may be because in CoS@NiS-80%, the ratio of Co to Ni is 1:4, which leads to the majority of the NiS phase in the catalyst.

As shown in Figure S1a, pure CoS has a hexagonal nanosheet structure with a thickness of about 31 nm, while CoS@NiS-20% also exhibits a hexagonal nanosheet structure, approximately 34.5 nm thick, as depicted in Figure 2b. In contrast, the pure NiS shows an irregular shape and obvious stacking and curling of nanosheets. As depicted in Figure S1b, its thickness is indistinct. When the Ni-to-Co ratio is 1:1, CoS@NiS-50% exhibits morphological characteristics of NiS (Figure S1c). CoS@NiS-80% has extremely irregular edges, and its average thickness reaches the minimum value of all samples, which is about 18 nm (Figure 2c). Previous research reported that the abundant crystal base of 2D transition metal dichalcogenides is catalytically inert. Instead, its HER activity mainly comes from its unsaturated edges and defects [48]. Figure 2d and Figure S2 show the SEM images of CoS@NiS-80% after a 100 h OER stability test. The nanosheets become extremely irregular, with agglomeration and curling occurring among them. Extremely irregular edges in nanomaterials usually have a higher defect density. This can significantly affect the electronic structure and surface reactivity of the materials, thereby improving their catalytic performance. In addition, it can increase the specific surface area and the exposure of active sites [49]. However, agglomeration and curling among nanosheets indicate that the nanosheet structure of the catalyst is destroyed to some extent during the long-term reaction. Furthermore, agglomeration of nanosheets during the long-term reaction usually leads to a significant decrease in specific surface area and in the exposure of active sites. Besides the aggravation of edge irregularity and the stacking of nanosheets, it is obvious that a large number of holes with uneven diameters appear on the nanosheets. These morphological changes indicate significant surface reconstruction during the prolonged OER process. The morphological changes of CoS@NiS-80% can be more significant after a stability test in alkaline seawater (Figure S3). CoS@NiS-80% agglomerates into irregular particles in a large area with the complete disappearance of the nanosheet structure, making the active surface area of CoS@NiS-80% and the exposure degree of active sites decrease significantly. In the elemental mapping images, Co, Ni and S elements are uniformly distributed in the CoS@NiS-80%, and the atomic ratio of Co, Ni and S is approximately equal to the ratio of raw materials, which confirms the successful synthesis of CoS@NiS-80% catalyst (Figure 2e–h and Figure S4). However, after a 100-h stability test, EDS results show that the quantitative fractions of Ni, Co and S are 71.14%, 17.25% and 11.61%, respectively. The Ni-to-Co ratio still remains about 4:1. But a large amount of the S element has been dissolved (Figure S5). This situation can also be found in CoS@NiS-80% after the alkaline seawater stability test. For CoS@NiS-80%, after a stability test in the alkaline seawater, the quantitative fractions of Ni, Co, S and Cl are 58.58%, 13.34%, 27.48% and 0.6%, respectively. Although the alkaline seawater stability test duration is much shorter than 100 h, a large amount of S element is still dissolved. The Ni-to-Co ratio deviates from 4:1 (Figure S6). The relative content of Co atoms on the electrode decreases. In brief, no matter what system it is, it is clear that Co and Ni atoms will transform from metal sulfides to other species. It has been reported that transition metal compounds can transform into transition metal hydroxyl oxides during OER [40].

**Figure 3:**
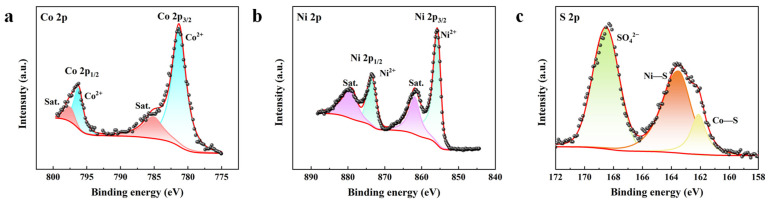
XPS spectra of CoS@NiS-80%. (**a**) Co 2p, (**b**) Ni 2p and (**c**) S 2p.

X-ray photoelectron spectroscopy (XPS) is used to further evaluate the composition and valence state of the CoS@NiS-80%. Figure 3 confirms the coexistence of the Co, Ni and S elements of CoS@NiS-80% before the electrochemical tests. In Figure 3a, the XPS spectrum of Co 2p, with peaks located at 781.3 eV and 785.2 eV, can be attributed to Co^2+^ and satellite peaks [44,50,51]. The Ni 2p XPS spectrum in Figure 3b exhibits two peaks at 855.8 eV and 873.6 eV, which correspond to Ni 2p_3/2_ and Ni 2p_1/2_ of Ni^2+^. The remaining two peaks at 861.9 eV and 878.9 eV correspond to satellite peaks [52]. For the S 2p XPS spectrum in Figure 3c, the peaks at 162.1 eV and 163.5 eV correspond to the Co-S bond and Ni-S bond, respectively. And the peak at 168.5 eV originates from SO_4_^2−^, which may be caused by the partial oxidation of sulfur atoms [33,34,52]. The XPS results prove that Co and Ni atoms mainly exist as Co^2+^ and Ni^2+^ in the catalyst and indicate the successful synthesis of CoS@NiS-80%.

**Figure 4:**
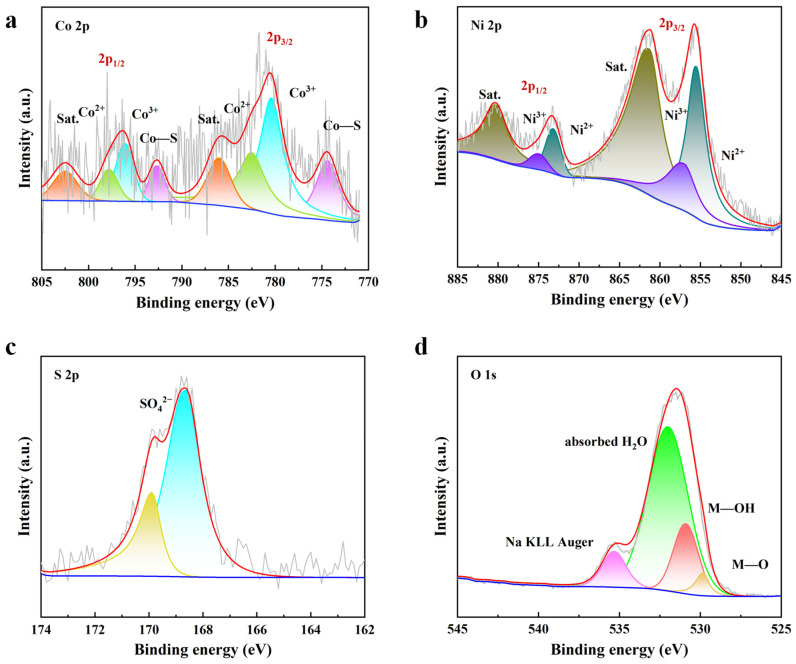
XPS spectra of CoS@NiS-80% after 100 h stability test in 1 M KOH. (**a**) Co 2p, (**b**) Ni 2p, (**c**) S 2p and (**d**) O 1s.

Furthermore, XPS is used to deeply analyze the changes in the substances and valence states of the elements in CoS@NiS-80% after 100 h of the OER stability test in 1M KOH (Figure 4). For the Co 2p spectrum (Figure 4a), the main peaks located at 780.4 eV and 796.1 eV can be indexed to Co^3+^, and peaks located at 782.5 eV and 797.8 eV correspond to Co^2+^ [36]. Peaks at 774.4 eV and 792.7 eV can be ascribed to the Co-S bond, while the remaining two at 786 eV and 802.5 eV correspond to satellite peaks [53]. The Ni 2p spectrum (Figure 4b) shows that the main peaks located at 855.6 eV and 873.3 eV can be ascribed to Ni^2+^, while the peaks located at 857.5 eV and 875.1 eV are related to Ni^3+^ [52]. The remaining two peaks are indexed to satellite peaks. The S 2p spectrum is shown in Figure 4c. It is obvious that the original sulfur–metal bond in Figure 3c disappears. Peaks at 168.6 eV and 169.9 eV can be assigned to the high-oxidation-state sulfur species [54]. The above results indicate that during the prolonged process of OER, parts of Co^2+^ and Ni^2+^ are oxidized to Co^3+^ and Ni^3+^ with the disappearance of S^2−^. Under high OER overpotential, S^2−^ ions on the surface are probably oxidized to soluble SO_x_^δ−^ during OER. And then SO_x_^δ−^ dissolves in electrolytes, exposing divalent metal ions (Co^2+^ and Ni^2+^) to a strong alkaline environment. Thus, EDS results show the massive dissolution of sulfur ions. Subsequently, Co^2+^ and Ni^2+^ ions may directly combine with hydroxyl groups to produce Co(OH)_2_ and Ni(OH)_2_ with low OER activity. However, part of Co(OH)_2_ and Ni(OH)_2_ may be oxidized in situ to CoOOH and NiOOH, with excellent OER activity under high OER overpotential. The O 1s spectrum of CoS@NiS-80% after 100 h of the OER process (Figure 4d) can prove this speculation. In the O 1s spectrum, peaks located at 529.8 eV, 530.9 eV and 532.0 eV correspond to M-O, M-OH and adsorbed H_2_O, respectively [55]. Regarding the peak located at 535 eV, it can be ascribed to Na KLL Auger. The coexistence of M-O and M-OH indicates the formation of NiOOH and CoOOH. In addition, the CoS@NiS-80% after the stability test in alkaline seawater is also analyzed by XPS (Figure S7). The result is similar to that shown in Figure 4.

### 3.2. Electrochemical Characterizations of Catalysts

**Figure 5:**
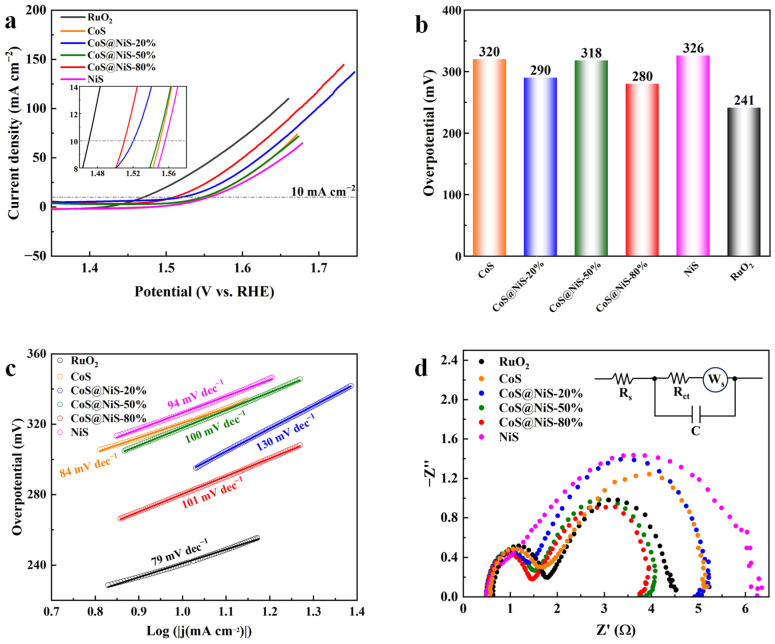
(**a**) The OER LSV curves with 85% iR compensation of samples (the internal diagram is an enlarged diagram of OER LSV curve near the current density of 10 mA cm^−2^). (**b**) The overpotential at current density of 10 mA cm^−2^ for different electrocatalysts. (**c**) Tafel plots of different samples. (**d**) The EIS results of electrocatalysts at the overpotential corresponding to their current density of 10 mA cm^−2^, respectively.

The investigation of OER performance for the CoS@NiS-x samples is tested in 1 M KOH. Before the scanning LSV test, all catalysts are subjected to 20 cycles of cyclic voltammetry (CV). The voltage range of CV is −0.8~0.2 V vs. Hg/HgO, and the CV results can be seen in Figure S8. To avoid the influence of the oxidation peak on the experimental results, the LSV curves scans from high potential to low potential. As shown in Figure 5a,b, CoS displays an overpotential of 320 mV at the current density of 10 mA cm^−2^, which is much higher than the overpotential of commercial RuO_2_ (240 mV). For pure NiS, the OER overpotential at 10 mA cm^−2^ is 326 mV. The results of pure NiS and CoS demonstrate their low OER activity. However, after CoS coupled with NiS, the OER activity of CoS@NiS-x samples exhibited an obvious increase. The overpotential of CoS@NiS-20% at 10 mA cm^−2^ is 290 mV, which is 30 mV lower than CoS and 36 mV lower than NiS, respectively. Additionally, CoS@NiS-50% needs 318 mV to achieve 10 mA cm^−2^. More notably, the overpotential of CoS@NiS-80% is 280 mV to drive 10 mA cm^−2^, which is 40 mV lower than pure CoS and only 40 mV higher than RuO_2_. An obvious trend is that in the composite samples, the OER activity first decreases and then increases with the increase of NiS content. The Tafel slope can be used to explain the OER reaction kinetics. The Tafel slope of the samples is shown in Figure 5c. The Tafel slope of CoS@NiS-80% is 101 mV dec^−1^ higher than CoS@NiS-50% (100 mV dec^−1^) and RuO_2_ (79 mV dec^−1^) but lower than CoS@NiS-20% (130 mV dec^−1^). The Tafel slopes of pure CoS and NiS are 84 mV dec^−1^ and 94 mV dec^−1^, respectively.

**Figure 6:**
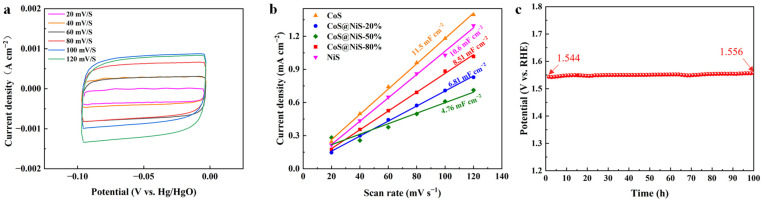
(**a**) CV curves of CoS@NiS-80% at the scan rates of 20, 40, 60, 80, 100 and 120 mV s^−1^. (**b**) C_dl_ curves of catalysts. (**c**) The chronopotentiometric curves of catalysts at the current density of 10 mA cm^−2^ in 1 M KOH.

Apart from the overpotential and Tafel slope, EIS and C_dl_ are also employed to further study the OER activity of the samples. Figure 5d exhibits that the CoS@NiS-80% has a smaller charge transfer resistance (3.25 Ω) than CoS@NiS-20% (4.43 Ω), CoS@NiS-50% (3.30 Ω), RuO_2_ (3.88 Ω), CoS (4.53 Ω) and NiS (5.74 Ω). The EIS results indicate that the interface coupling between CoS and NiS could enhance the charge transfer ability between adjacent phases and improve the conductivity of the catalyst, thus accelerating the charge transfer in the catalytic process. Electric double-layer capacitance is employed to further explore the OER performance of the CoS@NiS-80% heterostructure. As observed in Figure 6a,b, the CoS@NiS-80% has the highest C_dl_ value (8.51 mF cm^−2^) as compared to CoS@NiS-20% (6.81 mF cm^−2^) and CoS@NiS-50% (4.76 mF cm^−2^). CoS and NiS exhibited C_dl_ values of 11.5 mF cm^−2^ and 10.6 mF cm^−2^, respectively. Therefore, the CoS@NiS-80% presents the largest ECSA value (212.75) among CoS@NiS-x, which explains the reason for its best OER performance. Figure 6c shows the chronoamperometric stability plots when the current density is 10 mA cm^−2^. Durability is another crucial parameter for OER catalysts. CoS@NiS-80% displayed excellent long-time stability. At the current density of 10 mA cm^−2^, it worked continuously for 100 h, where the OER performance can still maintain 96.2%. The above electrochemical test results show that CoS@NiS-80% exhibits good OER catalytic activity and excellent stability. A comparative table is summed to evaluate the OER performance of CoS@NiS-80% against other similar electrocatalysts (Table 1).

**Figure 7:**
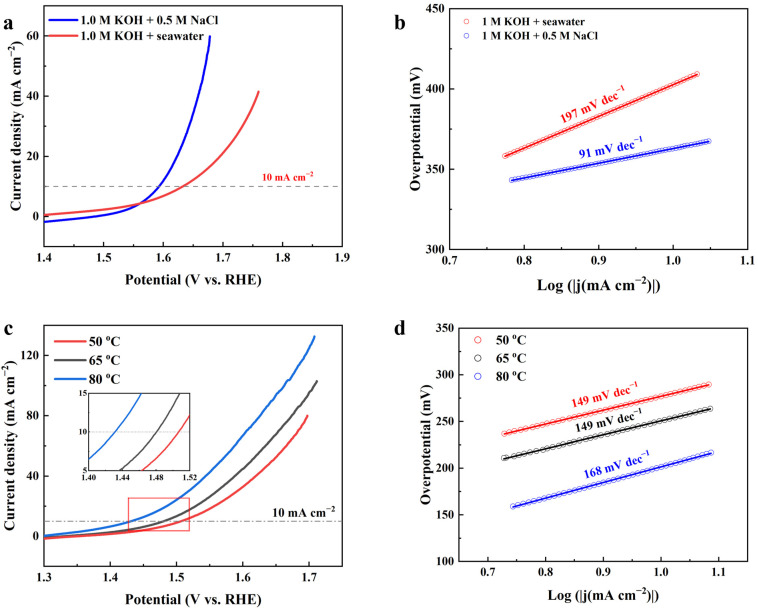
(**a**,**b**) The OER LSV curves with 85% iR compensation and Tafel plots of CoS@NiS-80% in alkaline-simulated seawater and alkaline seawater electrolytes. (**c**,**d**) The OER LSV curves with 85% iR compensation (the internal diagram is an enlarged diagram of OER LSV curve near the current density of 10 mA cm^−2^) and Tafel plots of CoS@NiS-80% in alkaline-simulated seawater and alkaline seawater electrolytes at different temperatures.

The OER performance of CoS@NiS-x catalysts is also investigated in different environments, including alkaline-simulated seawater (1.0 M KOH + 0.5 M NaCl), alkaline seawater (1.0 M KOH + seawater) and 1.0 M KOH solution at different temperatures. As shown in Figure 7a, in an alkaline-simulated seawater and alkaline seawater electrolytes, the OER performance of CoS@NiS-80% decreases significantly. And the potential window of the alkaline-simulated seawater (pH = 14.20) for OER is 1.301 V vs. RHE (Figure S9a), which is close to 1.302 V vs. RHE (Figure S9b) in the 1 M KOH (pH = 14.23). The required overpotential of CoS@NiS-80% at 10 mA cm^−2^ is 363 mV in the 1 M KOH + 0.5 M NaCl. In the 1 M KOH + seawater (pH = 14.31), the potential window of the electrolyte for OER is 1.307 V vs. RHE (Figure S9c), which is higher than the value (1.302 V vs. RHE) in 1 M KOH (pH = 14.23). And the overpotential is 403 mV at 10 mA cm^−2^. According to a previous study, the existence of chloride ions (Cl^−^) will cause electrode corrosion, damaging the surface and structure of catalysts. Additionally, compared with hydroxyl (OH^−^), the electronegativity of chloride ion is weaker than that of hydroxide ion, which means that chlorine evolution/oxidation reaction (ClER/ClOR) will compete with anodic OER in the catalytic process. Nevertheless, there are insoluble by-products in the 1 M KOH + seawater. These insoluble by-products like Mg(OH)_2_ will block the active sites of catalysts, hindering the OER process [56]. The above factors also affect the reaction kinetics of the catalyst. The Tafel slope of CoS@NiS-80% in the alkaline-simulated seawater is 91 mV dec^−1^. However, in the alkaline seawater, the Tafel slope of CoS@NiS-80% increases sharply, which is 197 mV dec^−1^ (Figure 7b). The stability test of CoS@NiS-80% in the alkaline-simulated seawater and alkaline seawater is also carried out. Figure S10a shows the chronoamperometric stability plots when the current density is 10 mA cm^−2^. In the alkaline-simulated seawater, the CoS@NiS-80% can still work stably for over 100 h. However, the stability of CoS@NiS-80% in the alkaline seawater is obviously worse than that in 1 M KOH and the alkaline-simulated seawater (Figure S10b). The result shows that in the alkaline seawater, the OER catalytic activity of CoS@NiS-80% continuously decreases within 10 h. Finally, the overpotential at 10 mA cm^−2^ rises to about 486 mV (the initial overpotential is 370 mV). From the perspective of thermodynamics, the increase of temperature is conducive to the decrease of Gibbs free energy in the process of OER, which is more conducive to the spontaneous progress of OER. According to the Nernst equation, the equilibrium potential of OER reaction will also decrease with the increase of temperature. It is predicted that CoS@NiS-80% will exhibit better OER activity at 50 °C, 65 °C and 80 °C than RT. Especially at 80 °C, CoS@NiS-80% achieves the lowest overpotential of 201 mV at 10 mA cm^−2^, which is lower than at RT (280 mV), 50 °C (277 mV) and 65 °C (251 mV) (Figure 7c). The Tafel slope of CoS@NiS-80% is 149 mV dec^−1^, 149 mV dec^−1^ and 168 mV dec^−1^ at 50 °C, 65 °C and 80 °C, respectively (100.87 mV dec^−1^ at RT) (Figure 7d).

**Figure 8:**
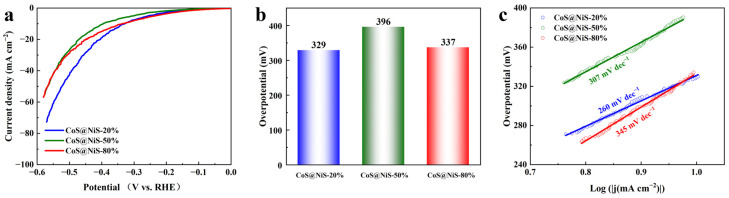
(**a**) The HER LSV curves with 85% iR compensation of samples. (**b**) The overpotential at current density of 10 mA cm^−2^ for different electrocatalysts. (**c**) Tafel plots of different samples.

Furthermore, the HER performance of CoS@NiS-x catalysts is exhibited in Figure 8a,b. CoS@NiS-20% displayed the lowest HER overpotential of 329 mV at 10 mA cm^−2^. CoS@NiS-50% had the highest overpotential of 396 mV at 10 mA cm^−2^. Although CoS@NiS-80% displayed the best OER performance among CoS@NiS-x catalysts, its HER performance was not as ideal as its OER performance, as 337 mV overpotential was needed to achieve 10 mA cm^−2^. The Tafel slope is shown in Figure 8c. Although CoS@NiS-20% has the best HER activity among CoS@NiS-x catalyst, its Tafel slope was the lowest, which was 259.6 mV dec^−1^. The Tafel slope of CoS@NiS-80% was 344.7 mV dec^−1^, and CoS@NiS-50%’ s Tafel slope was 306.7 mV dec^−1^.

Based on the above discussion and previous studies, the OER performance of CoS @ NiS-80% is attributed to the synergistic effect of heterogeneous interface and surface reconstruction. The nanosheet structure of CoS@NiS-80% exposes more CoS/NiS heterogeneous interfaces for OER. It is reported that when CoS and NiS form a heterogeneous interface, electron transfer will occur spontaneously at the interface due to the Fermi energy level difference. In CoS@NiS, electrons transfer from NiS to CoS, which increases the electron density of the Co site [57,58]. On the one hand, the electron coupling effect between different components enhances the electron conductivity, thus reducing the charge transfer resistance of the catalyst [59]. On the other hand, increasing the electron density of the Co site optimizes the *OOH adsorption and decreases the OER energy barrier in the Co site. Additionally, lattice mismatch often exists at heterogeneous interfaces, which induces sulfur vacancies or the coordination of unsaturated Co sites, and these defects can be directly used as OER active sites [60]. Surface reconstruction is a common dynamic phenomenon in the electrocatalytic process of non-noble metal catalysts. It has significant effects on the performance of cobalt sulfide-based OER catalysts [53]. In the process of surface reconstruction, Co^2+^ is gradually and irreversibly oxidized to high-valence Co^3+^/Co^4+^. Previously, it was reported that the adsorption free energy of the Co^3+^ site of CoOOH for the *OH/*O/*OOH intermediate was closer to the peak of the volcanic map, which was significantly better than the original CoS [61]. The S ion is oxidized to soluble sulfate (SO_4_^2−^). Sulfur loss leads to surface roughening and increases the active area [62]. However, it may also lead to the peeling of the active layer. Reasonable heterostructure, such as the core–shell structure, inhibits the excessive dissolution of active species through the interface [63,64]. Electron transfer between heterogeneous interfaces may accelerate the generation of active species at the interface. With the interface effect and synergistic effect, the performance of the CoS@NiS-x catalyst is further improved.

## 4. Conclusions

In this work, we successfully developed CoS@NiS-80% nanosheet electrocatalyst via a simple hydrothermal method. With the nanosheet structure, interface effect, synergistic effect between CoS and NiS and the effect of surface reconstruction, CoS@NiS-80% shows good OER performance in 1 M KOH. At the current density of 10 mA cm^−2^, the OER overpotential of CoS@NiS-80% is 280 mV with a Tafel slope of 100.87 mV dec^−1^. In addition, CoS@NiS-80% can work stably at the current density of 10 mA cm^−2^ for 100 h, where the OER performance of it can still maintain 96.2%. Furthermore, at 80 °C, CoS@NiS-80% exhibits better OER activity, with an overpotential of 201 mV to achieve 10 mA cm^−2^. In alkaline-simulated seawater and alkaline seawater environments, the overpotential of CoS@NiS-80% at 10 mA cm^−2^ is 363 and 402 mV, respectively. This work can provide a new idea for constructing efficient transition metal chalcogenide catalysts by heterogeneous interface engineering and surface reconstruction.

## Figures and Tables

**Table 1:** Comparison of the OER performance of the CoS@NiS-80% catalyst with similar electrocatalysts.

Catalysts	Current Density j (mA cm^−2^)	Overpotential at Corresponding j (mV)	Tafel Slope (mV dec^−1^)	Stability (h)	Refs.
CoS-Ag-3%	10	293	55.3	30	[20]
CoS/rGO	10	290	71	24	[53]
CoS/MoS_2_	10	281	79	12	[13]
Co-MoS_2_	10	312	126.07	100	[14]
MoS_2_/NiS	100	400	181	30	[38]
Mo-CoS_2_/NC	10	296	65	40	[24]
MXene@CoS/FeS_2_	10	278	52.7	40	[2]
CoS@NiS-80%	10	280	101	100	This work

## Data Availability

The original contributions presented in this study are included in the article/Supplementary Material. Further inquiries can be directed to the corresponding authors.

## Supplementary Materials

The following supporting information can be downloaded at: https://www.mdpi.com/article/10.3390/nano15161216/s1, Figure S1: (a) The SEM images of pure CoS. (b) The SEM images of pure NiS. (c) The SEM images of CoS@NiS-50%; Figure S2: The SEM image of CoS@NiS-80% after 100 h OER stability test; Figure S3: (a, b) The SEM image of CoS@NiS-80% after 10 h OER stability test in alkaline seawater electrolyte; Figure S4: EDS images of CoS@NiS-80% before electrochemical tests; Figure S5: (a–d) Elemental mapping images of CoS@NiS-80% after 100 h stability test in 1 M KOH. Purple represents nickel, yellow represents sulfur and orange represents cobalt. (e) EDS images of CoS@NiS-80% after 100 h stability test in 1 M KOH; Figure S6: (a–e) Elemental mapping images of CoS@NiS-80% after stability test in the seawater. (f) EDS images of CoS@NiS-80% after stability test in the seawater; Figure S7: XPS spectra of CoS@NiS-80% after stability test in the seawater; Figure S8: CV curves of (a) CoS, (b) CoS@NiS-20%, (c) CoS@NiS-50%, (d) CoS@NiS-80% and (e) NiS (a) Ni 2p, (b) Co 2p, (c) S 2p and (d) O 1s; Figure S9: OER LSV curve of CoS@NiS-80% with scanning speed of 1 mV s^−1^ (85% iR compensation) in different electrolyte. (a) 1 M KOH. (b) 1 M KOH + 0.5 M NaCl. (c) 1 M KOH + seawater; Figure S10: The chronopotentiometric curves of CoS@NiS-x at the current density of 10 mA cm^−2^ in (a) alkaline-simulated seawater stability test and (b) alkaline seawater stability test.
