# Supplementary material for: Facile Engineering of CoS@NiS Heterostructures for Efficient Oxygen Evolution Reaction
Source: Nanomaterials (Basel). 2025 Aug 8;15(16):1216. doi: 10.3390/nano15161216 (PMC12388220; doi:10.3390/nano15161216)
Supplement: Supplementary file 1 [file nanomaterials-15-01216-s001.zip › nanomaterials-3760020-supplementary.pdf]

Supporting Information

Facile engineering of CoS@NiS heterostructures for efficient oxygen evolution reaction

Ting Yang[1], Aiyi Dong[2,*], Weimin Liao[3], Xun Zhang[1], Yinhua Ma[2,*], Li Che[2], Honglin Gao[3,*]

[1] Transportation Engineering College, Dalian Maritime University, Dalian 116026, China; yangting@dlmu.edu.cn (T.Y.); zx2220223740@dlmu.edu.cn (X.Z.)
[2] School of Science, Dalian Maritime University, Dalian 116026, China; liche@dlmu.edu.cn (L.C.)
[3] Marine Engineering College, Dalian Maritime University, Dalian 116026, China; lwm20021202@dlmu.edu.cn (W.L.)
* Correspondence: aiyidong@dlmu.edu.cn (A.D.); mayh@dlmu.edu.cn (Y.M); honglin_gao@dlmu.edu.cn (H.G.)

Experimental section

1.1 Synthesis of NiS nanosheets

1.25 mmol of $Ni(NO_3)_2 \cdot 6H_2O$ and 5 mmol of urea were dissolved in 17.5 mL of ethylene glycol and 2.5 mL of deionized water, and a uniform emerald green solution was formed by stirring. Then the obtained emerald green solution was transferred to a stainless steel autoclave lined with PTFE and kept at 120 ℃ for 4 hours. Then, the obtained dark green $Ni(OH)_2$ nanosheets were collected by centrifugation, and directly immersed in 30 mL of 1 mol $L^{-1}Na_2S$ solution for 13 hours, and stirred at 500 rpm. Finally, the black product was collected by centrifugation, then washed with deionized water several times and dried in a vacuum environment of 50℃ for 17 hours.

1.2 Synthesis of CoS@NiS-x nanosheets

1.25·x (x=20%, 50%, 80%) mmol of $Ni(NO_3)_2 \cdot 6H_2O$ and 1.25·(1-x) mmol of $Co(NO_3)_2 \cdot 6H_2O$ were dissolved in 17.5 mL of ethylene glycol and 2.5 mL of deionized water, and a uniform solution was formed by stirring. Then the obtained solution was transferred to a stainless steel autoclave lined with PTFE and kept at 120 ℃ for 4 hours. Then, the hydroxide nanosheets were obtained by centrifugation. Subsequently, the hydroxide was directly immersed in 30 ml of 1 mol L^{-1} Na_2S solution for 13 hours and stirred at 500 rpm. Ultimately, centrifugation was used to collected the black product, followed by washing with deionized water several times, and dried in a vacuum environment of 50 ℃ for 17 hours.

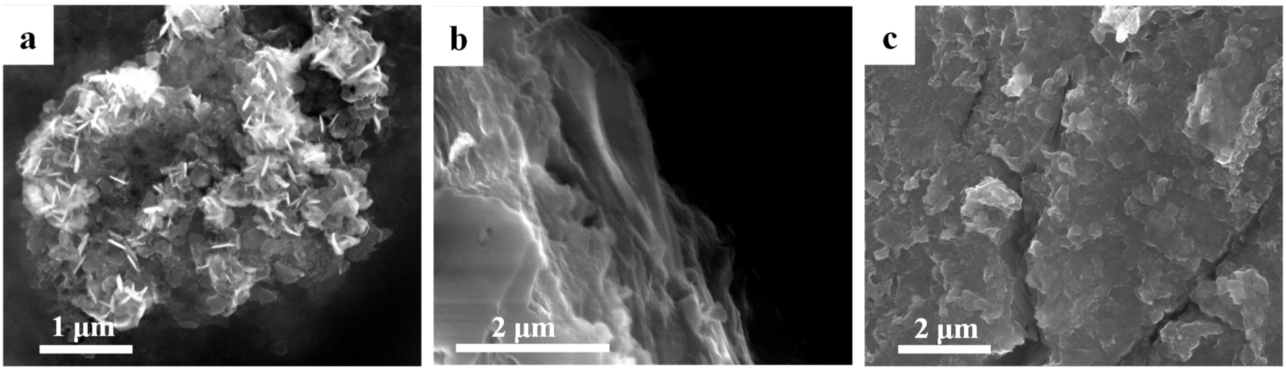

Figure S1. (a) The SEM images of pure CoS. (b) The SEM images of pure NiS. (c) The SEM images of CoS@NiS-50%.

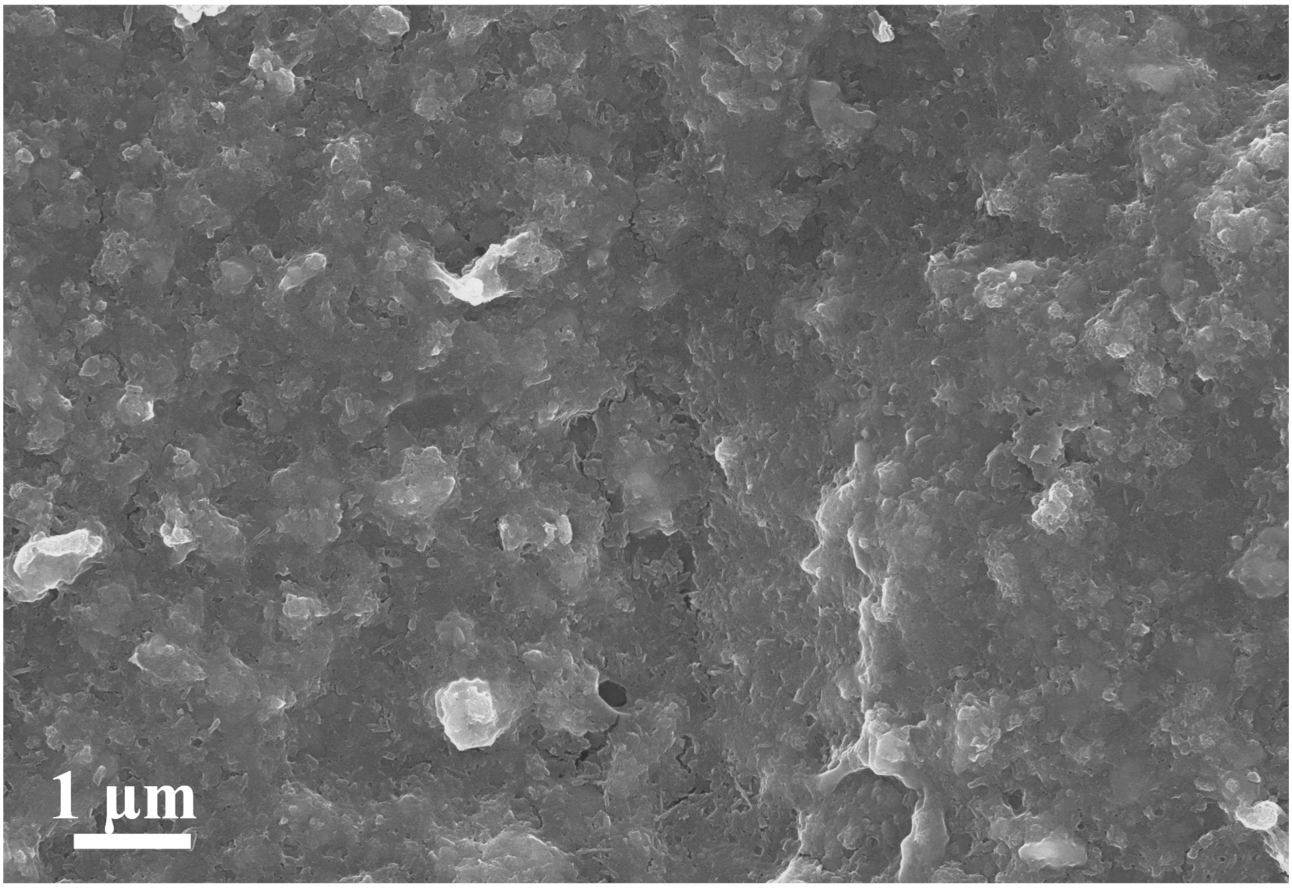

Figure S2. The SEM image of CoS@NiS-80% after 100 hours OER stability test.

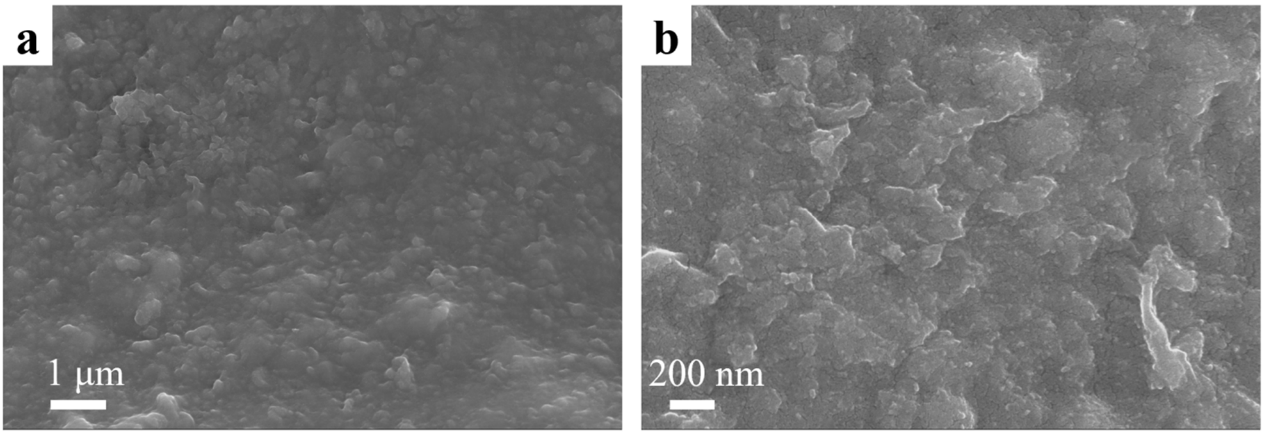

Figure S3. (a, b) The SEM image of CoS@NiS-80% after 10 hours OER stability test in alkaline seawater electrolyte.

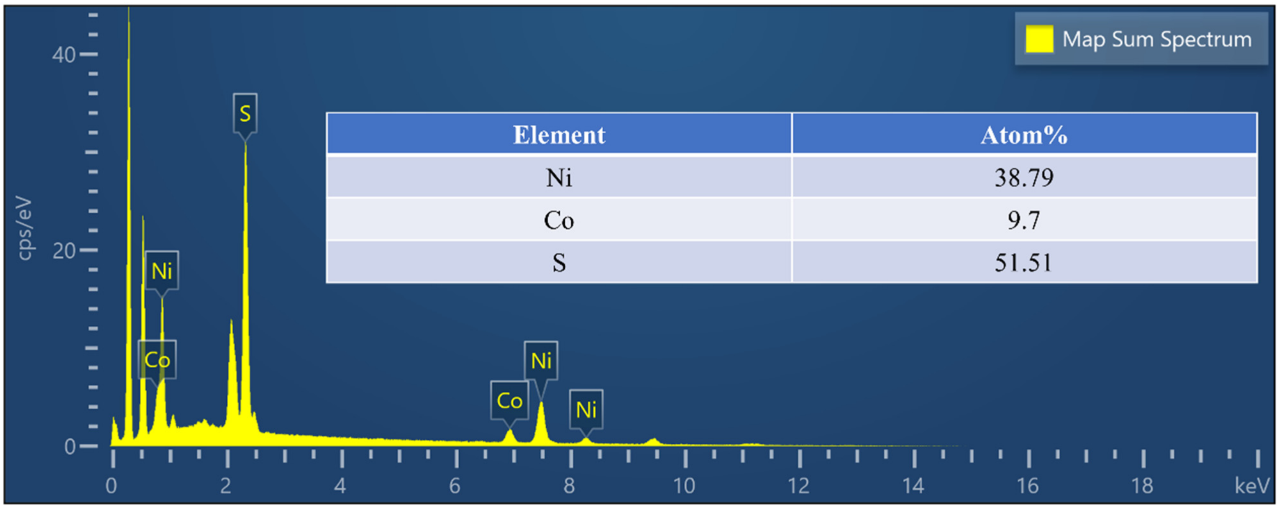

Element	Atom%
Ni	38.79
Co	9.7
S	51.51

Figure S4. EDS images of CoS@NiS-80% before electrochemical tests.

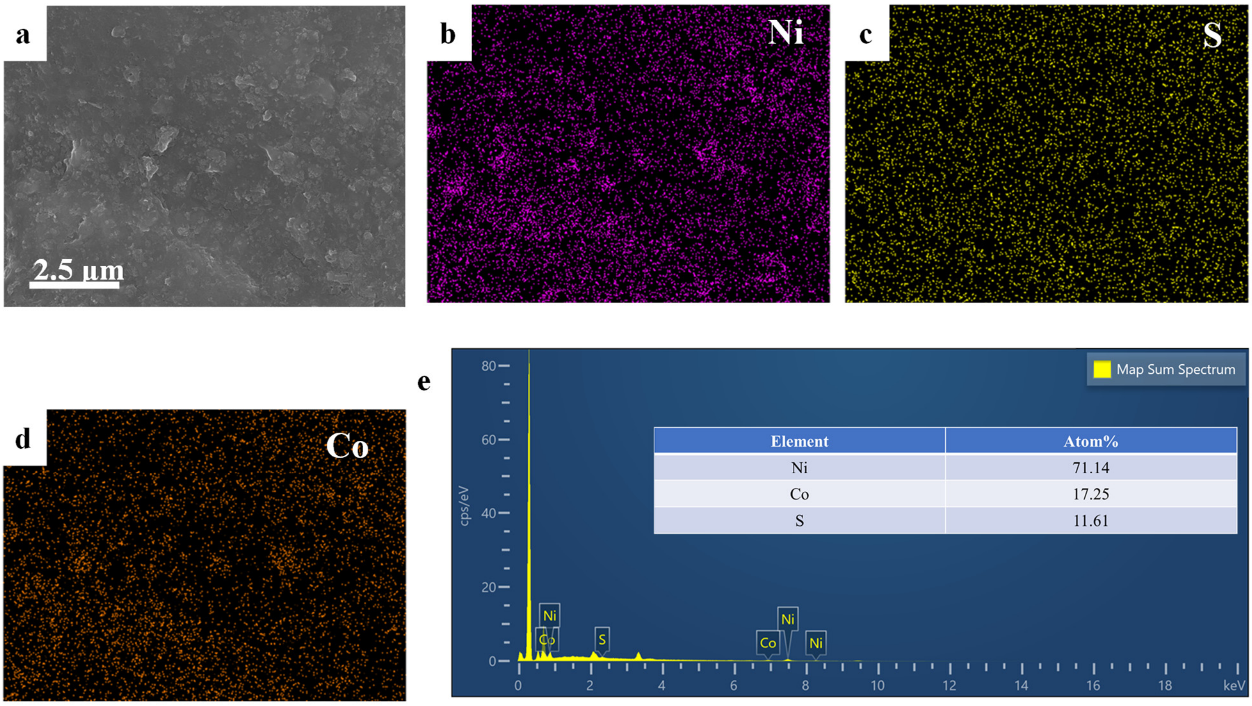

Figure S5. (a-d) Elemental mapping images of CoS@NiS-80% after 100 hours stability test in 1 M KOH. Purple represents nickel, yellow represents sulfur and orange represents cobalt. (e) EDS images of CoS@NiS-80% after 100 hours stability test in 1 M KOH.

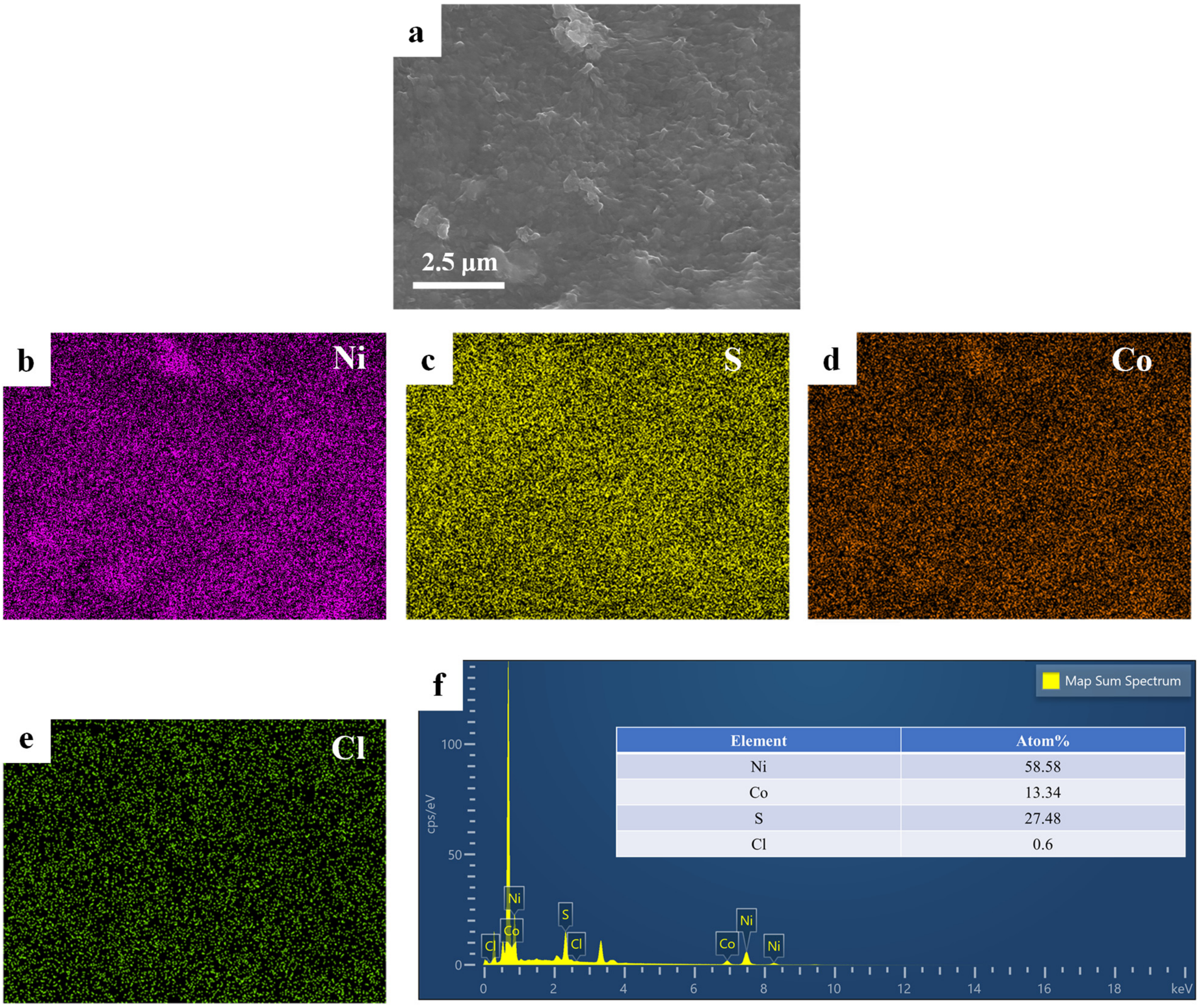

Element	Atom%
Ni	58.58
Co	13.34
S	27.48
Cl	0.6

Figure S6. (a-e) Elemental mapping images of CoS@NiS-80% after stability test in the seawater. (f) EDS images of CoS@NiS-80% after stability test in the seawater.

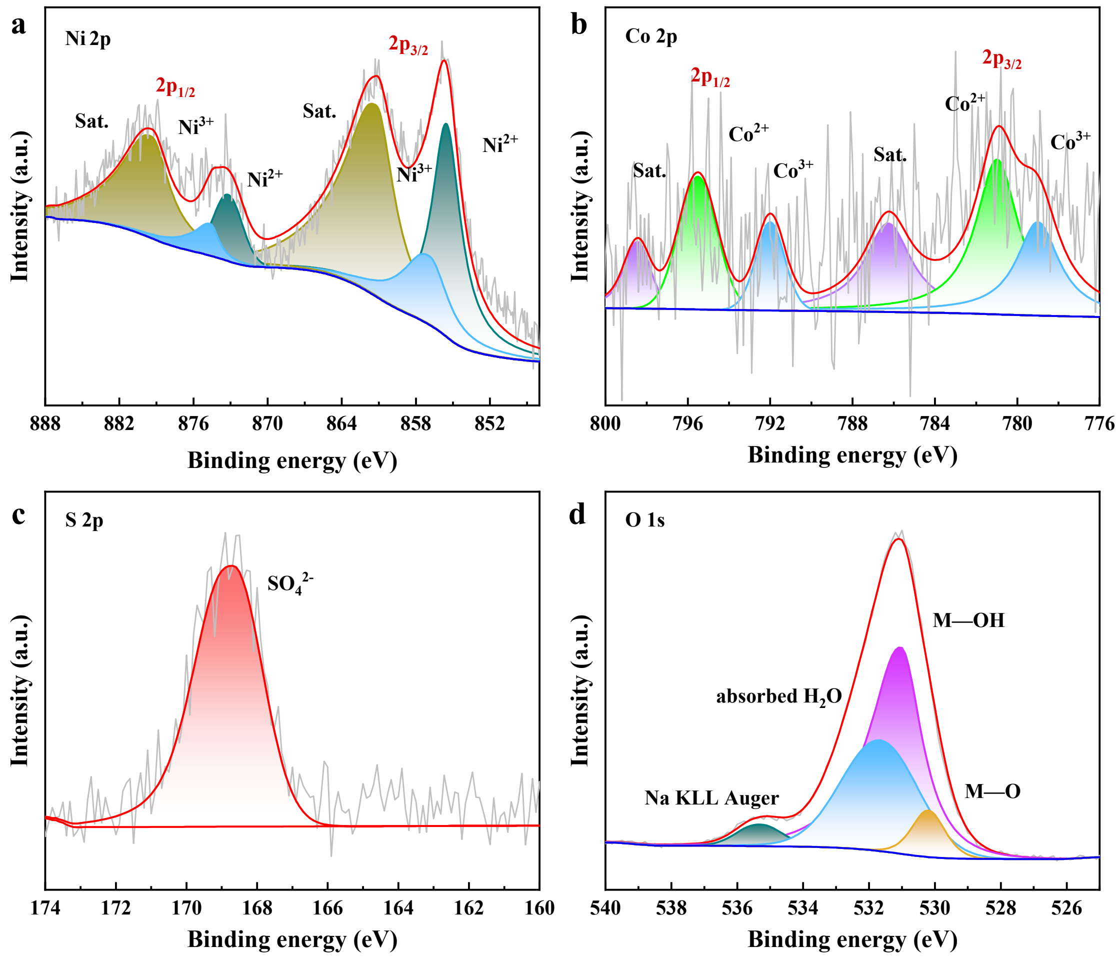

Figure S7. XPS spectra of CoS@NiS-80% after stability test in the seawater. (a) Ni 2p, (b) Co 2p, (c) S 2p and (d) O 1s.

For Ni 2p spectrum (**Figure S7(a)**), peaks located at 855.5 eV and 873.2 eV can be ascribed to Ni^{2+}. Peaks located at 874.6 eV and 856.9 eV can correspond to Ni^{3+}. The rest peaks located at 861.2 eV and 879.4 eV are satellite peaks [1]. **Figure S7(b)** exhibits the Co 2p spectrum. Peaks located at 779 eV and 792 eV are related to Co^{3+}. Peaks located at 781.0 eV can be ascribed to Co^{2+}. The other two peaks located at 786.3 eV and 798.5 eV are satellite peaks [2]. For the S 2p spectrum (**Figure S7(c)**), the main peak located at 168.7 eV corresponds to SO_4^{2-}[1]. As for O 1s spectrum (**Figure S7(d)**), peaks located at 530.2 eV and 531 eV are M-O and M-OH bond. The XPS results indicate S ions are oxidized to SO_4^{2-} and then dissolved in electrolyte. Subsequently metal atoms are partially oxidized with the generation of transition metal oxyhydroxide [3].

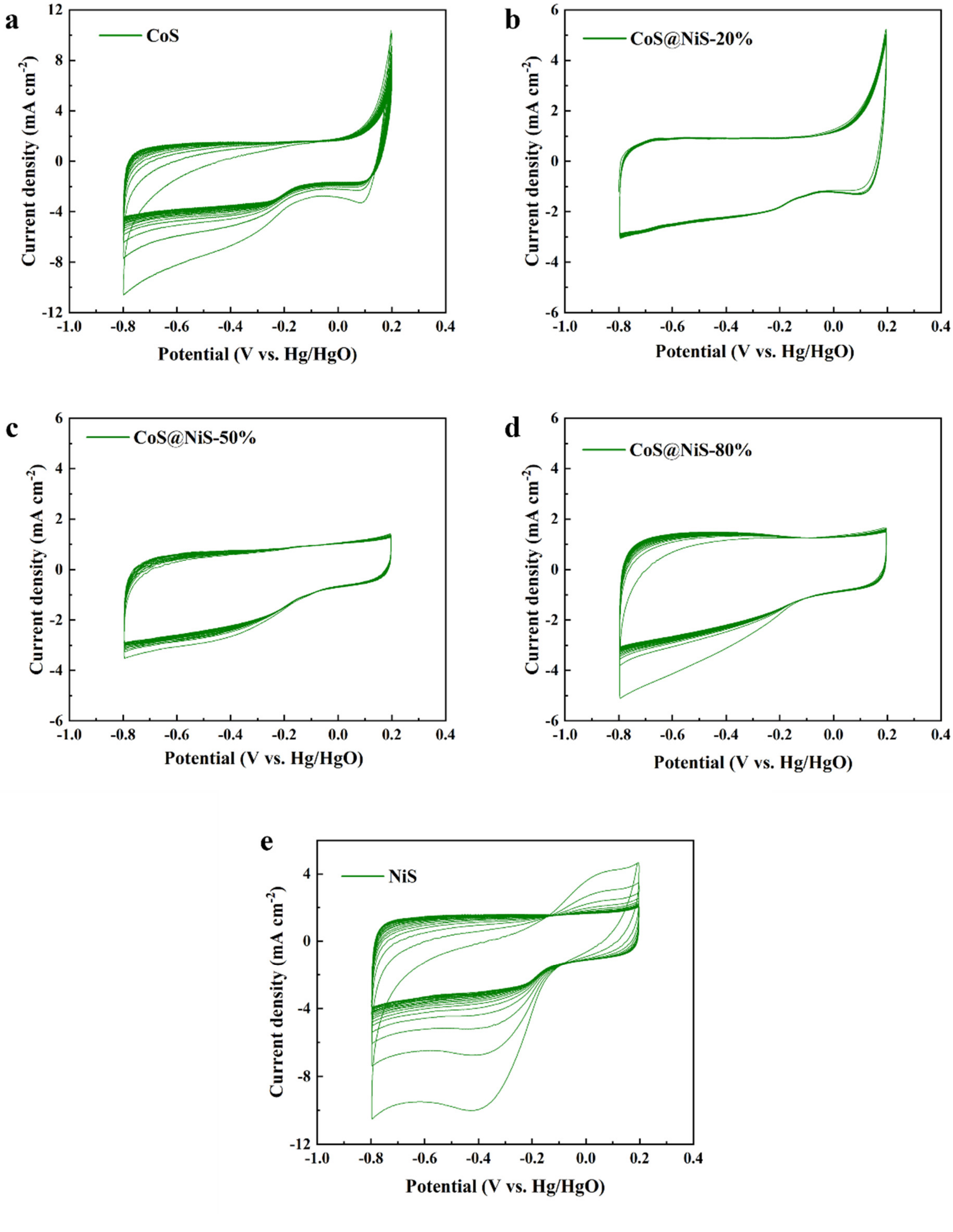

Figure S8. CV curves of (a) CoS, (b) CoS@NiS-20%, (c) CoS@NiS-50%, (d) CoS@NiS-80% and (e) NiS.

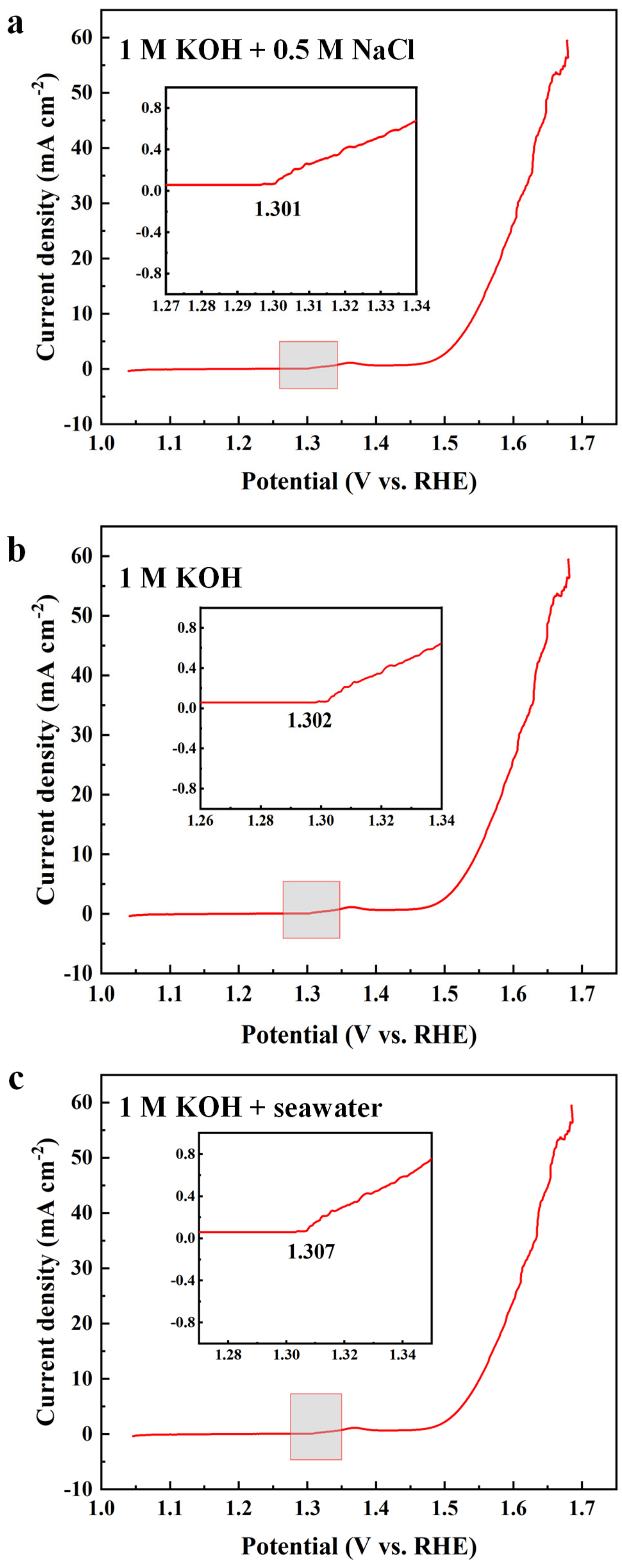

Figure S9. OER LSV curve of CoS@NiS-80% with scanning speed of 1 mV s^{-1} (85% iR compensation) in different electrolyte. (a) 1 M KOH. (b) 1 M KOH + 0.5 M NaCl. (c) 1 M KOH + seawater.

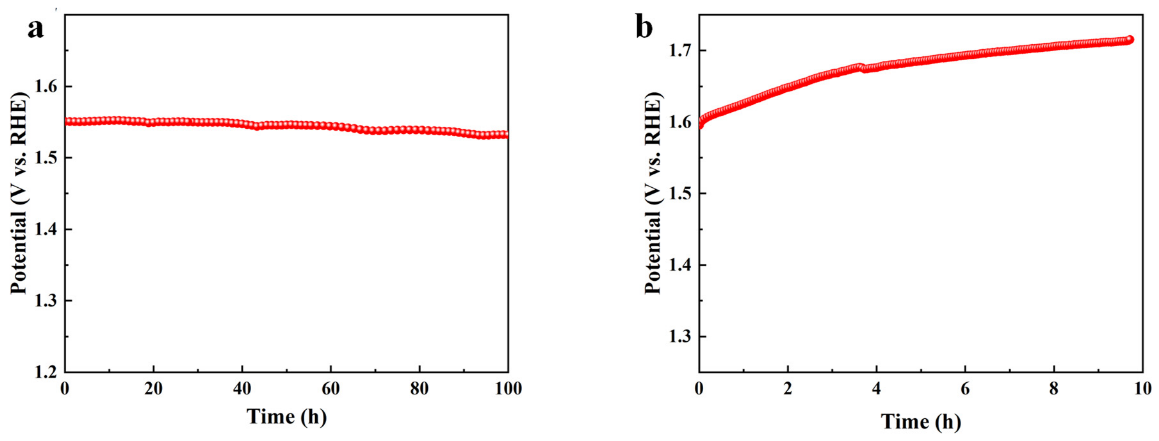

Figure S10. The chronopotentiometric curves of CoS@NiS-x at the current density of 10 mA cm^{-2} in (a) alkaline simulated seawater stability test and (b) alkaline seawater stability test.

1. Wang, F.;Zhu, Y.;Tian, W.;Lv, X.;Zhang, H.;Hu, Z.;Zhang, Y.;Ji, J., and Jiang, W., Co-Doped Ni_3S_2@CNT Arrays Anchored on Graphite Foam with a Hierarchical Conductive Network for High-Performance Supercapacitors and Hydrogen Evolution Electrodes. *J. Mater. Chem. A* **2018**, *6*, 10490-10496. https://doi.org/10.1039/c8ta03131b
2. Luo, W.;Yu, Y.;Wu, Y.;Wang, W.;Jiang, Y.;Shen, W.;He, R.;Su, W., and Li, M., Strong Interface Coupling Enables Stability of Amorphous Meta-Stable State in CoS/Ni_3S_2 for Efficient Oxygen Evolution. *Small* **2024**, *20*, 2310387. https://doi.org/10.1002/smll.202310387
3. Li, S.;Li, Y.;Zhang, J.;Liu, X.;Zhang, K.;Zhang, Y., and Song, X.-m., Charge Separation at $BiVO_4$/Co_3O_4 and $BiVO_4$/CoOOH Interfaces: Differences between Dense and Permeable Cocatalysts. *Appl. Surf. Sci.* **2023**, *624*, 156965. https://doi.org/10.1016/j.apsusc.2023.156965
